# Staying sane in psychiatric residency during COVID times

**DOI:** 10.1192/bji.2023.2

**Published:** 2023-05

**Authors:** Maryam Ayub

**Affiliations:** Postgraduate Psychiatry Resident, Academic Department of Psychiatry and Behavioral Sciences, King Edward Medical University, Lahore, Pakistan. Email: drmaryamayub@yahoo.com

**Keywords:** Suicide, education and training, social functioning, low- and middle-income countries, stigma and discrimination

## Abstract

This article gives a junior psychiatry resident's personal story of burnout during the COVID-19 pandemic: what led to it, what helped her get through it and the continual process of working to avoid burnout in the future.

Burnout is characterised by depersonalisation, emotional exhaustion and a feeling of decreased personal achievements that causes a decline in professional efficacy.^1^ Psychiatrists have been found to be at higher risk of substance misuse, depression, suicide and dysfunctional behaviour than other medical professionals.^2,3^ Higher depression scores among psychiatrists seem to be followed by higher suicide rates.^4^ In a study of psychiatry residents during the COVID-19 pandemic, burnout symptoms were found in 27.3% and another 27.3% reported symptoms of depression. In addition, 16.5% reported having both burnout and depressive symptoms, with a significant relationship between the two. Participants in the first 2 years of training who had received mental health treatment in the previous 2 years were at higher risk.^5^ In 2020, healthcare workers around the world were affected by the unprecedented COVID-19 pandemic in a number of ways, including increased workload, limited access to personal protective equipment, a lack of resources that could save lives, frustration, isolation and fear of possible infection in themselves and their families.^6^ A study of 10 178 postgraduate medical trainees in Pakistan reported that the prevalence of depressive symptoms was 26.4% and that of anxiety symptoms was 22.6% following the COVID-19 outbreak.^7^

This article is about my personal story of burnout, what led up to it, what helped me get through it and the ongoing measures I take to avoid going to that place again. It was sometime in June 2020 on an ordinary workday (or as ordinary as it could be during those times), I entered a meeting room and I realised I didn't care enough about the meeting because nothing good could come out of it. And I didn't care enough for my life because nothing good could come out of that either and maybe in that moment I should just die. As a person who has always loved her life and her choices, I realised in less than a minute that I was not in the right mindset if I had started thinking like that. I went into the admin office and took a blank piece of paper, thought of my resignation, which I planned to write by the end of the day, and went back to work, realising the relief the thought of quitting my job had brought and I no longer wanted to quit my life. I have come to realise that those few seconds or minutes and that blank paper saved my life. This was something private and known only to my friends and family, but working on suicide and self-harm-related research, I now realise that the stories in which people made it past burnout and depression are really important, so finally I am sharing mine.

## The lead up

Aside from the obvious pandemic, there was first the absolute lack of patient interaction. As a second year resident who had gone into psychiatry for patient interaction, the switch to online and telemedicine alone was something of an occupational shock. Add to that random roster duties – after a year of treating patients, I was not seeing any get better because there were no regular duties. I was living with my 80-year-old grandmother. The thought that I could bring the disease home to my family by going everyday to hospital was nauseating. I had little emotional support available outside my immediate family because of lockdowns and social distancing. Each day the hospital management were just turning out notices of extra duties in COVID intensive care units (ICUs). As a psychiatry trainee with minimum experience in medicine, the thought that I could cause someone's death because of lack of adequate supervision was horrifying. Add to that the lack of personal protective equipment, full-time telemedicine duties, COVID-related research that was springing up everywhere and the online seminars on giving psychological first aid to first responders and protecting the mental health of doctors: we psychiatric trainees could feel ourselves drowning. I remember talking to couple of my colleagues after conducting a seminar on burnout and suicide help for doctors and we were laughing because we were going through such burnout ourselves and no one was helping us. Administrators everywhere in the world might have been doing this but in my opinion all the bureaucracy and the show of ‘everything is great’ was particularly high in my work setting. We were being forced to do extra duties without any pay, organise COVID-related seminars and pretend that everything was fine. It was obviously not. I had myself completed a burnout questionnaire few weeks before and, apart from suicidal thoughts (which I didn't have at that time), I had scored really high. Again as psychiatric trainees we were expected to heal our physician colleagues, forgetting the fact that for junior psychiatric residents this was a lot for them to take in.

## What helped me get through it?

Among the things helped me (apart from that blank piece of paper, which I kept in my purse for several months so I could write my resignation any time I felt like I should give up), the most important were my colleagues. One colleague, now a dear friend, realised I was going through a lot that week, and even though she was away with her family, she sent me a box of brownies. That box and the small card with it made me tear up at the time. Then there were the handful of seniors who realised that we were being put through things that no one could survive without psychological damage. Just the acknowledgement itself helped. The research I had worked on was published in a high-impact-factor journal, and my consultant, seeing the work I had put in, made me third author even though I had worked with a ‘last author’ credit in mind. One of my senior consultants post-pandemic told us that he himself had talked to a psychiatrist friend when he was overburdened. I wish he had told us that during the awful time because we wouldn't have felt so alone and overwhelmed.

I consider myself lucky with ICU duties, because I got into a medical unit in which I always had physician colleagues/friends and seniors available to discuss the patients. One of my psychiatric trainee colleagues wasn't so lucky and she went through similar burnout before going to the hospital administration because she felt she was letting the patients down. Also, we eventually received personal protective equipment (some provided by the administration, some we bought ourselves when it became available). The lockdowns relaxed a few weeks later and that helped my morale because I could finally be at ease with the fact that I didn't feel all alone. I started talking to my friends and family regularly. I took a week off and went for a vacation with my sister and a friend once the COVID wave was over. It helped me learn that I should work proactively before burnout sets in.

## Not going to that place again

Now I take time out before I am close to the brink instead of waiting for burnout to happen. I speak up when I see a bad call from consultants that can affect the mental health of my juniors. My colleagues and I are making sure that we are there for our juniors, especially during their first 2 years, when everything can be overwhelming. The loss of social connectedness has been shown to play a major role in physician burnout. It is vital that leaders follow organisational and teamwork guidelines for prevention and recovery by dedicating time every day to creating a work environment and culture that encourages a sense of belonging, where every staff member feels appreciated and part of a collaborative mission to improve the health and well-being of patients and colleagues.^8^ Burnout prevention can be promoted actively by encouraging respect and politeness, identifying and treating the shame and guilt that may come with errors of commission and omission, and swiftly stepping in to stop humiliation at the hands of superiors. Other interventions include Schwartz Rounds, which provide a structured multidisciplinary forum in which staff (clinical and non-clinical) and medical students can candidly discuss interpersonal and emotional issues that they face when caring for patients and families, developing long-term relationships through peer support and mentoring, providing space and time to meet and share experiences, and more.^9^

## Data Availability

Data availability is not applicable to this article as no new data were created or analysed in this study.
